# Clinical and biochemical signs of polycystic ovary syndrome in young women born preterm

**DOI:** 10.1530/EJE-20-1462

**Published:** 2021-06-03

**Authors:** Marika Paalanne, Marja Vääräsmäki, Sanna Mustaniemi, Marjaana Tikanmäki, Karoliina Wehkalampi, Hanna-Maria Matinolli, Johan Eriksson, Marjo-Riitta Järvelin, Laure Morin-Papunen, Eero Kajantie

**Affiliations:** 1 Finnish Institute for Health and Welfare, Population Health Unit, Oulu and Helsinki, Finland; 2 PEDEGO Research Unit (Research Unit for Pediatrics, Dermatology, Clinical Genetics, Obstetrics, and Gynecology), Medical Research Center Oulu (MRC Oulu), Oulu University Hospital and University of Oulu, Oulu, Finland; 3 Children’s Hospital, Helsinki University Hospital and University of Helsinki, Helsinki, Finland; 4 Research Center for Child Psychiatry, University of Turku, Turku, Finland; 5 INVEST Research Flagship, University of Turku, Turku, Finland; 6 Folkhälsan Research Center, Helsinki, Finland; 7 Department of General Practice and Primary Care, University of Helsinki and Helsinki University Hospital, Helsinki, Finland; 8 Department of Obstetrics and Gynecology, National University of Singapore, Yong Loo Lin School of Medicine, Singapore, Singapore; 9 Singapore Institute for Clinical Sciences, Agency for Science, Technology, and Research, Singapore, Singapore; 10 Imperial College, London, UK; 11 Department of Clinical and Molecular Medicine, Norwegian University of Science and Technology, Trondheim, Norway

## Abstract

**Objective:**

It has been suggested that adverse early life exposures increase the risk of developing polycystic ovary syndrome (PCOS) in later life. We hypothesized that women born preterm would have more biochemical and clinical signs of PCOS than women born at term.

**Design:**

The ESTER Preterm Birth Study participants were born in Northern Finland and identified from the Northern Finland Birth Cohort and the Finnish Medical Birth Register. Altogether, 74 women born very or moderately preterm (<34 gestational weeks, VMPT), 127 born late preterm (at 34–36 weeks, LPT), and 184 born full term (≥37 weeks, controls) were included in the analysis (mean age: 23.2 years).

**Methods:**

We measured serum total testosterone and sex hormone-binding globulin (SHBG) and calculated the free androgen index (FAI). PCOS according to the clinical and biochemical signs was defined either as hirsutism and oligoamenorrhea (via questionnaire) or as oligoamenorrhea and elevated testosterone levels (>2.4 nmol/L).

**Results:**

Women born VMPT/LPT exhibited 33.0% (8.7, 62.8)/16.4% (−2.0, 38.1) higher testosterone, 28.5% (5.3, 45.9)/24.1% (5.6, 38.9) lower SHBG levels, and 64.6% (19.4, 127.1)/42.5% (11.1, 82.9) higher FAI than controls after adjusting for age and recruitment cohort, maternal BMI, smoking, and pregnancy disorders, parental education, history of hypertension, diabetes, myocardial infarction or stroke, and subject’s birth weight s.d. Odds ratios for having PCOS were 1.67 (0.44, 6.23)/3.11 (1.26, 7.70).

**Conclusions:**

Women born preterm have a more hyperandrogenic hormonal profile, and those born LPT are approximately three times more likely at risk to have PCOS compared to women born at term.

## Introduction

There is increasing evidence that adults born with very low birth weight (<1500 g, VLBW) or very preterm (<32 gestational weeks, GW) show enhanced cardiometabolic risk factors including elevated blood pressure (1, 2) and lower insulin sensitivity (1, 3, 4). Some of these risks are also present in the large group of young adults born late preterm (34–36 GW), which is consistent with a dose-response relationship between the degree of prematurity and non-optimal metabolic characteristics such as higher body fat percentage and higher waist circumference, lower insulin sensitivity, and elevated risk of metabolic syndrome (5).

The metabolic characteristics that are associated with an increased risk of metabolic syndrome, type 2 diabetes, and cardiovascular disease include hyperandrogenism and polycystic ovary syndrome (PCOS). PCOS is one of the most common endocrine disorders; it affects about 5–18% of women worldwide depending on ethnicity and on the criteria applied (6, 7, 8).

It has been suggested that the fetal environment, such as intrauterine growth retardation, might program individuals for the development of PCOS later in life (9, 10). For example, some retrospective studies have shown that girls born small for gestational age (SGA) are at a higher risk of developing early pubarche, early menarche, and PCOS (11, 12), but published results have been controversial (13). Women born preterm with VLBW had similar reproductive hormone levels than their peers born at term in the Swedish cohort (14). However, women born < 32 weeks had elevated levels of adrenal androgens (DHEA sulfate and androstenedione), particularly when born SGA (15). The associations of preterm birth with PCOS in women born preterm (<37 weeks) have been studied only a little, even though 11% of all babies, about 14.9 million annually worldwide, are born preterm (16).

We hypothesized that women born preterm present more clinical and biochemical signs of PCOS than women born at term.

## Subjects and methods

The participants come from the ESTER Preterm Birth Study, including 753 young adults identified from the Northern Finland Birth Cohort (NFBC, born 1985–1986) or from the Finnish Medical Birth Register (FMBR, born 1987–1989 in the same area) who participated in a clinical study in 2009–2011 and had confirmed gestational ages (5). Of them, 394 (52.3%) were women. After excluding nine women who reported cerebral palsy, mental disability, and/or other severe physical disability, 74 women born very or moderately preterm (VMPT, born before 34 GW), 127 born late preterm (LPT, born at 34–36 GW), and 184 born full-term (controls, born ≥37 GW) were included in the analysis. In addition, those who were pregnant (*n* = 18) were excluded from the analysis of weight, BMI, body fat percentage, sex hormone-binding globulin (SHBG), and testosterone. Details of the nonparticipation analysis have been published previously (5).

### Perinatal data

The perinatal data from the participants recruited from the NFBC came from the cohort database, originally collected from medical records. We collected corresponding data from those invited through the FMBR from their hospital and maternal welfare clinic records. We independently confirmed the length of gestation (17) and diagnoses of maternal gestational diabetes (GDM), hypertension (gestational or chronic), or pre-eclampsia (including superimposed) according to prevailing criteria by reviewing their original hospital records. We calculated birth weight standard deviation (s.d.) scores according to Finnish birth weight standards (18). We defined 'small for gestational age' as more than 2 s.d. below the mean for sex and length of gestation.

### Clinical examination

The subjects attending the clinic between 7:00 and 9:00 h were examined by two trained study nurses. Height (cm) was measured three times. Waist circumference (cm), measured midway between the lowest rib and the iliac crest, was measured twice.The mean height and weight were calculated. We used a segmental multi-frequency bioelectrical impedance (InBody 3.0, Biospace Co., Seoul, Korea) to assess weight (kg) and body composition, including fat mass (kg) and percentage of body fat (%). BMI was calculated (kg/m^2^).

Blood samples were collected after overnight fasting and 2 h after a 75 g oral glucose load. Serum insulin (µU/mL) was measured at three points (0, 60, and 120 min). Serum total testosterone (nmol/L) and SHBG (nmol/L) concentrations were measured to assess hyperandrogenemia using an Architect ci8200 analyzer (Abbott Laboratories) with 2nd generation chemiluminescent microparticle immunoassay, which has been developed to improve accuracy at low concentrations among women and shows similar discriminatory power for PCOS as liquid chromatography-mass spectrometry, the reference method (19) (Abbott Laboratories) at the Biochemistry laboratory of the Genetic and Biomarker Unit in the National Institute for Health and Welfare. According to the reference values of the reagent manufacturer, testosterone levels > 2.4 nmol/L were considered elevated. The homeostatic model assessment of insulin resistance (HOMA-IR) and free androgen index (FAI) were calculated: FAI = (testosterone/SHBG) × 100 (20).

The participants completed questionnaires covering their medical histories and medications (including use of oral contraceptives), family medical history, socioeconomic status, and lifestyles. Socioeconomic status was assessed as the educational attainment of the more highly educated parent and was categorized in four levels (dummy coded). Self-reported physical activity levels were converted to total metabolic equivalent hours per week (METh/wk) (21).

### Definition of PCOS according to the clinical and biochemical signs

Several questions were asked to assess hirsutism, a clinical manifestation of androgen excess: 'Do you have excessive growth of hair? Do you remove facial hair regularly (excluding the eyebrows)? How many times per month?' Hirsutism was considered present if a woman had excessive growth of hair or removed facial hair regularly at least four times per month. To assess menstrual cycle irregularity, one question inquiring about the minimum and maximum length and another inquiring about the regularity of the menstrual cycle without hormonal contraception was asked: 'Is your menstrual cycle often (more than twice per year) longer than 35 days?' (22). Oligomenorrhea was defined if the participants answered 'yes' to the aforementioned question, and irregular menses were defined if the length of the menstrual cycle regularly varied over 7 days or if the participant had been treated for irregular menstrual cycle.

According to the Rotterdam definition and the International guidelines, the diagnostic criteria of PCOS require women to have two out of three of the following manifestations: (i) oligo- or anovulation, (ii) clinical and/or biochemical signs of hyperandrogenism, and (iii) polycystic ovaries at ultrasound (23, 24). As the ultrasound of the ovaries had not been performed in the study population, the group referred in the present study as ' women with PCOS' included the participants having (i) oligo- or anovulation and hirsutism or (ii) oligo- or anovulation and elevated testosterone levels (>2.4 nmol/L).

### Ethics

The research protocol was approved by the Coordinating Ethics Committee at Helsinki and Uusimaa Hospital District, and all participants provided written informed consent.

### Statistical analysis

The data were analyzed using SPSS 22.0. Crude differences between groups were assessed with Dunnett’s *t*-test and Pearson’s chi-square test. Differences in continuous and categorical variables were assessed with linear and logistic regression.

The main outcome variables of the study are testosterone, SHBG and FAI levels and PCOS according to the clinical and biochemical signs. Regression Model 1 included age and source cohort (NFBC or FMBR). Model 2 comprises variables in Model 1 and parental and prenatal confounding factors including parental educational attainment as a proxy for childhood socioeconomic position, maternal BMI, smoking, hypertension, pre-eclampsia and GDM during pregnancy, and subject’s birth weight. s.d. scores were used as indicators of fetal conditions during pregnancy, and parental history of hypertension, diabetes, and myocardial infarction or stroke was used as proxies for genetic susceptibility. Model 3 includes both the confounders and mediators and it comprises variables in Model 2 and current characteristics, including body fat percentage, physical activity, smoking, and hormonal contraception. We did not adjust the results with the phase of the menstrual cycle, because it has been shown that there is only a low intraindividual variability of SHBG over the menstrual cycle (25). We conducted an additional analysis by excluding those born SGA to ensure that the association between preterm birth and hyperandrogenism did not depend on intrauterine growth retardation.

## Results

Perinatal and neonatal characteristics of subjects and educational and medical history of parents are described in Table 1.

**Table 1:** Perinatal and neonatal characteristics and educational attainment and medical history of parents of women born very or moderately preterm (VMPT), late preterm (LPT), or full term (controls). Data are presented as mean ± s.d. or *n* (%).

	VMPT, *n* = 74		LPT, *n* = 127		Controls, *n* = 184
	Values	*P*-value^a^	Values	*P*-value^a^	
Gestational age, weeks	31.9 ± 2.0	<0.001	35.8 ± 0.8	<0.001	40.0 ± 1.3
Birth weight, g	1680 ± 450	<0.001	2646 ± 554	<0.001	3494 ± 456
Birth weight SDS	−1.1 ± 1.3	<0.001	−0.6 ± 1.4	<0.001	0.0 ± 1.0
Small for gestational age	17 (23.0%)	<0.001	17 (13.4%)	<0.001	4 (2.2%)
Multiple pregnancy	17 (23.0%)	<0.001	16 (12.6%)	<0.001	4 (2.2%)
Maternal hypertension	11 (14.9%)	0.485	15 (12.1%)	0.024	21 (11.7%)
Maternal pre-eclampsia	23 (31.1%)	<0.001	14 (11.3%)	0.188	8 (4.4%)
Maternal gestational diabetes	2 (3.1%)	0.492	5 (4.2%)	0.342	3 (1.7%)
Maternal smoking during pregnancy	11 (15.7%)	0.855	26 (21.0%)	<0.001	30 (16.7%)
Cesarean section	49 (66.2%)	<0.001	35 (27.6%)	<0.001	20 (10.9%)
Parental education		0.825		0.758	
Basic, less, or unknown	6 (8.1%)		8 (6.5%)		13 (7.1%)
Secondary	47 (63.5%)		77 (62.5%)		107 (58.5%)
Lower-level tertiary	8 (10.8%)		17 (13.8%)		23 (12.6%)
Upper-level tertiary	13 (17.6%)		21 (17.1%)		40 (21.9%)
Maternal history of hypertension	14 (18.9%)	0.399	21 (16.5%)	0.655	27 (14.7%)
Maternal history of diabetes	2 (2.7%)	0.431	10 (7.9%)	0.280	9 (4.9%)
Maternal history of MI or stroke	0 (0.0%)	N/A	2 (1.6%)	0.088	0 (0.0%)
Paternal history of hypertension	17 (23.0%)	0.209	17 (13.4%)	0.480	30 (16.3%)
Paternal history of diabetes	4 (5.4%)	0.114	12 (9.4%)	0.486	22 (12.0%)
Paternal history of MI or stroke	3 (4.1%)	0.916	5 (3.9%)	0.859	8 (4.3%)

^a^Pearson’s chi-square test for categorical variables and Dunnett’s *t*-tests in many-to-one comparisons for continuous variables, VMPT or LPT vs controls.

MI, myocardial infarction.

Women born VMPT and LPT were slightly younger but had similar BMIs as the controls (Table 2). Women born LPT had higher waist circumference, serum concentrations of fasting insulin, and 120 min insulin in oral glucose tolerance test and HOMA-IR than controls. The differences in insulin levels and HOMA-IR were not statistically significant when adjusted for covariates. Women born VMPT more often reported regular smoking.

**Table 2:** Characteristics of women who were born very or moderately preterm, late preterm, and full term (controls). Data are presented as mean ± s.d. or as *n* (%).

	VMPT, *n* = 74		LPT, *n* = 127		Controls,* n* = 184
	Values	*P*-value^a^	Values	*P*-value^a^	
Age mean, years	23.0 ± 1.3	<0.001	23.1 ± 1.2	<0.001	23.5 ± 1.1
Height, cm	163.3 ± 5.1	0.547	164.6 ± 5.7	0.761	163.9 ± 5.9
Weight, kg	62.4 ± 0.26	0.999	62.2 ± 0.19	0.200	60.7 ± 0.20
BMI, kg/m^2^	22.8 ± 0.24	0.725	22.4 ± 0.18	0.227	22.0 ± 0.19
WC, cm	77.3 ± 0.17	0.065	75.8 ± 0.12	0.014	73.6 ± 0.13
Body fat percentage	28.7 ± 0.30	0.313	27.1 ± 0.27	0.101	25.9 ± 0.31
Fasting insulin, µU/mL	7.66 ± 0.65	0.295	7.37 ± 0.60	0.025	6.74 ± 1.52
120 min insulin µU/mL	30.90 ± 0.85	0.071	30.84 ± 0.85	0.040	28.46 ± 1.88
HOMA-IR	0.99 ± 0.63	0.507	0.96 ± 0.60	0.037	0.88 ± 1.50
TS, nmol/L	1.44 ± 1.48	0.857	1.35 ± 1.44	0.864	1.00 ± 1.00
Elevated TS levels^b^	6 (8.8%)	0.128	3 (2.5%)	0.495	7 (4.0%)
SHBG, nmol/L	112.89 ± (2.32)	0.044	142.40 ± (2.38)	0.107	149.05 ± 2.39
Free androgen index	1.32 ± 2.57	0.313	1.15 ± 2.54	0.414	0.87 ± 2.74
Physical activity, METh/wk^*^	23.5 ± 12.7	0.145	24.9 ± 14.5	0.676	24.3 ± 11.9
Daily smoking	23 (31.1%)	0.046	26 (20.5%)	0.844	36 (19.6%)
Hormonal contraception	24 (32.4%)	0.244	45 (35.4%)	0.394	74 (40.2%)
Hirsutism	11 (15.7%)	0.386	20 (16.7%)	0.190	20 (11.4%)
Irregular menstrual cycle	34 (49.3%)	0.383	50 (42.7%)	0.587	68 (39.5%)
Menstrual cycle >35 days	11 (16.2%)	0.407	13 (11.2%)	0.351	17 (10.1%)
PCOS^c^	4 (5.4%)	0.530	16 (12.6%)	0.143	14 (7.6%)

^a^Pearson’s chi-square test for categorical variables and Dunnett’s *t*-test in many-to-one comparisons for continuous variables, VMPT or LPT vs controls; ^b^Testosterone > 2.4 nmol/L; ^c^PCOS according to biochemical and clinical signs; ^*^Self reported leisure time physical activity.

HOMA-IR, homeostatic model assessment of insulin resistance; LPT, late preterm; METh, metabolic equivalent task hours; PCOS polycystic ovary syndrome; SHBG, sex hormone binding globulin; VMPT very or moderately preterm; WC, waist circumference; TS, testosterone.

### Hyperandrogenemia

**Figure 1:**
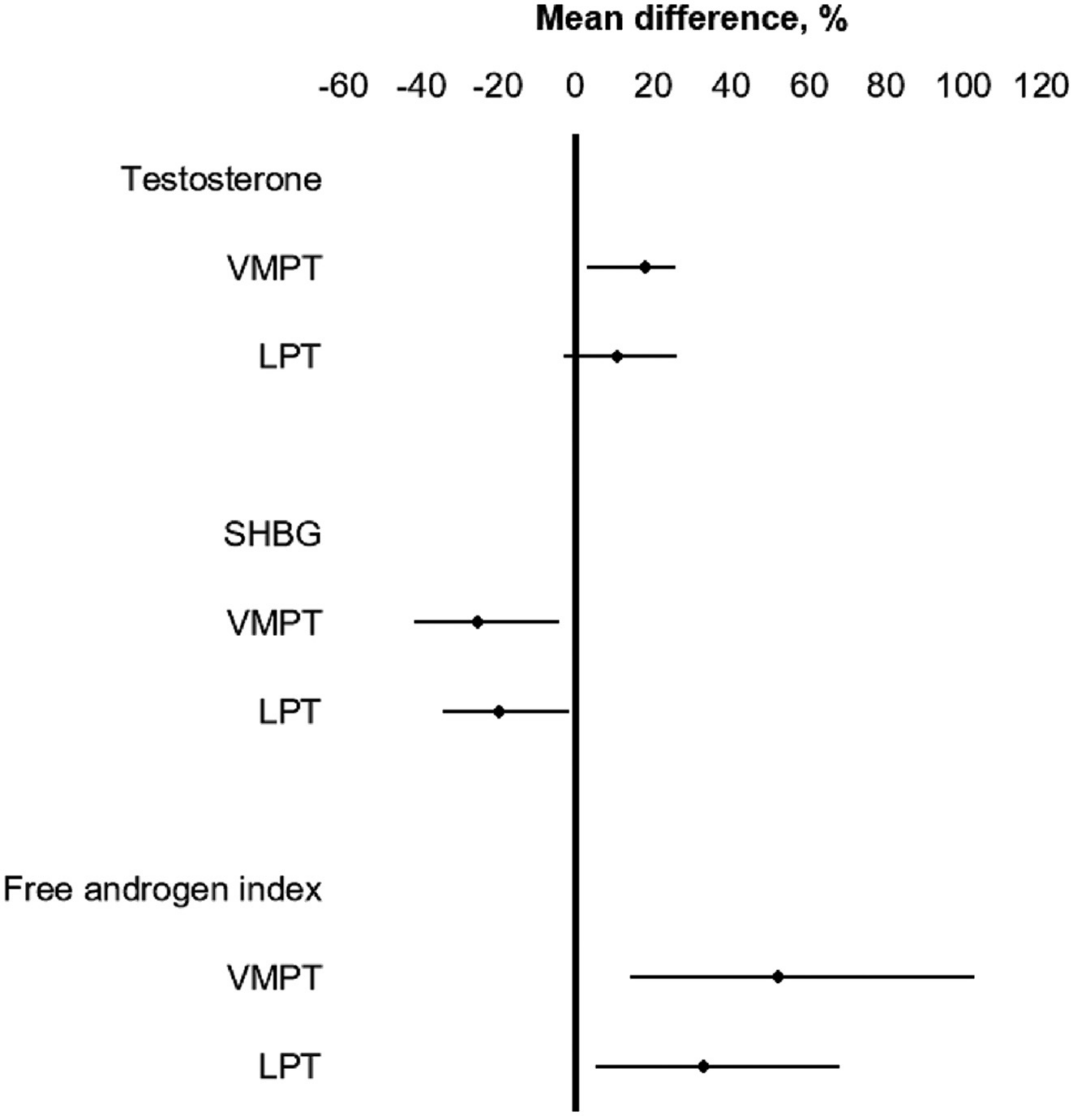
Mean differences and 95% CIs (error bars) in testosterone levels, sex hormone-binding globulin (SHBG) levels and free androgen index (FAI) in women born very or moderately preterm (VMPT) and late preterm (LPT) compared with controls (zero line) adjusted for age and recruitment cohort.

The mean absolute values of testosterone, SHBG and FAI are presented in Fig. 1

and Table 2. In the linear regression models, women born VMPT had 18.1% (95% CI: 3.0, 25.4) higher testosterone, 25.4% (95% CI: 4.4, 41.8) lower SHBG levels, and 52.1% (95% CI: 14.2, 102.7) higher FAI than women born at term after adjustment for age and recruitment cohort (Model 1, Table 3). Women born LPT had 10.5% (−3.1, 26.1) higher testosterone, and 19.6% (95% CI: 1.6, 34.4) lower SHBG levels, and 32.7% (95% CI: 5.0, 67.8) had higher FAI than their peers born at term (Model 1, Table 3). The differences were larger after adjusting for parental and prenatal factors (Model 2, Table 3). After adjusting for both parental and prenatal factors and current characteristics (Model 3, Table 3), the differences between women born VMPT and the controls increased regarding testosterone levels; they were attenuated regarding SHBG levels and FAI. However, with the same adjustments, the differences in SHBG levels and FAI between the LPT group and the controls remained similar (Model 3, Table 3).

**Table 3:** Mean differences (in percent) for sex hormone-binding globulin and testosterone, and odds ratios for self-reported polycystic ovary syndrome according to the clinical and biochemical signs with 95% CI in women born very or moderately preterm or late preterm compared to controls born full term.

Model	VMPT		LPT		Cases analyzed, *n*
	Values	*P*-value	Values	*P*-value	
Testosterone					
1	18.1% (3.0, 25.4)	0.017	10.5% (−3.1, 26.1)	0.136	347
2	33.0% (8.7, 62.8)	0.006	16.4% (−2.0, 38.1)	0.083	322
3	40.0% (7.7, 81.9)	0.012	16.7% (−5.3, 43.9)	0.148	312
SHBG					
1	−25.4% (−41.8, −4.3)	0.021	−19.6% (−34.4, −1.6)	0.035	347
2	−28.5% (−45.9, −5.3)	0.019	−24.1% (−38.9, −5.6)	0.013	316
3	−16.5% (−34.4, 6.2)	0.141	−18.9% (−32.5, −2.5)	0.026	212
FAI					
1	52.1% (14.2, 102.7)	0.004	32.7% (5.0, 67.8)	0.018	347
2	64.6% (19.4, 127.1)	0.002	42.5% (11.1, 82.9)	0.006	322
3	38.9% (5.5, 82.7)	0.019	31.5% (6.6, 62.3)	0.011	312
PCOS					
1	0.78 (0.24, 2.49)	0.67	2.00 (0.92, 4.30)	0.082	385
2	1.67 (0.44, 6.23)	0.45	3.11 (1.26, 7.70)	0.014	358
3	1.43 (0.34, 6.76)	0.65	4.24 (1.49, 12.10)	0.007	331

Covariates in linear and logistic regression models, 1: Age and recruitment cohort; 2: Variables in Model 1 and parental educational attainment, maternal BMI, smoking hypertension, pre-eclampsia and gestational diabetes during pregnancy; subject’s birth weight standard deviation scores and parental history of hypertension, diabetes, and myocardial infarction or stroke; 3: Variables in Model 2 and body fat percentage, physical activity, smoking, and hormonal contraception.

FAI, free androgen index; LPT, late preterm; OR, odds ratio; PCOS, polycystic ovary syndrome (according to clinical and biochemical signs); SHBG, sex hormone binding globulin; VMPT, very or moderately preterm.

When gestational age at birth was used as a continuous variable, a 1-week higher gestational age was associated with a 2.8% (95% CI: 0.1, 5.4) lower FAI (Model 1). The difference attenuated after adjusting for parental and prenatal factors (Model 2) and became statistically nonsignificant after adjusting for all covariates (Model 3). However, there was no statistically significant linear association between gestational age and total testosterone or SHBG level.

### PCOS according to clinical and biochemical signs

PCOS was present in 5.4% of the women in the VMPT group, 12.6% in the LPT group, and 7.6% in the control group. Odds ratio (OR) for PCOS was 0.78 (95% CI: 0.24, 2.49) in VMPT group and 0.67 (0.92, 4.39) (Model 1, Table 3). However, when adjusted for parental and prenatal confounding factors (Model 2), the ORs for PCOS were 1.67 (0.44, 6.23) for VMPT and 3.1 (1.26, 7.70) for LPT women. The ORs were higher after adjusting for all of the covariates (Model 3). We found no difference in the prevalence of PCOS between the VMPT group and the controls.

### The effects of prenatal factors

In linear regression models, a 1-unit-higher birth weight s.d. score predicted 4.5% (0.8%, 8.2%) higher total testosterone levels, 8.8% (0.7%, 16.3%) lower SHBG levels and 14.3% (4.1%, 25.5%) higher FAI (Model 2).

We conducted an additional analysis by excluding women born SGA. The mean difference in testosterone levels between the VMPT group and the controls increased after adjusting for age and recruitment cohort (Model 1) and attenuated when adjusted for parental and prenatal factors (Model 2); they were not statistically significant after adjusting all covariates (Model 3) compared to an analysis that included those born SGA. However, the differences in SHBG levels and FAI increased in both groups compared to controls after adjusting for Models 1–3; the ORs for PCOS were similar in all of the models.

Exposure to maternal GDM predicted 30.1% (95% CI: 9.3, 46.2) lower total testosterone and 49.7% (95% CI: 5.7, 73.2) lower SHBG levels (Model 2), but it did not have a statistically significant effect on FAI: 39.0% (95% −32.6, 186.3). Also, maternal history of type 1 or 2 diabetes predicted PCOS (OR: 6.8, 95% CI: 1.5, 31.9). We conducted an additional analysis by excluding the women exposed to maternal hypertension during pregnancy, pre-eclampsia, and GDM. The results were similar, and any differences became slightly larger. However, maternal hypertensive pregnancy disorders did not predict hyperandrogenism.

### Associations with body composition and hormonal contraception

In linear regression models, a 1% higher body fat percentage was associated with 0.5% (0.0, 1.1) higher total testosterone, 1.7% (95% CI: 0.6, 2.9) lower SHBG and 1.9% (95% CI: 0.8, 2.9) higher FAI levels. Use of hormonal contraception was associated with 15.0% (95% CI: 7.8, 21.6) lower total testosterone levels, 159.3% (95% CI: 119.2, 206.8) higher SHBG levels, and 68.0% (95% CI: 62.5, 72.6) lower FAI (Model 3). When hormonal contraception was not a covariate in Model 3, the difference between the preterm and control groups decreased regarding testosterone levels, but it increased regarding SHBG and FAI levels. The ORs for PCOS remained similar. We also performed a sensitivity analysis excluding the 154 women (40.0%) who used hormonal contraception. Differences in testosterone and FAI attenuated, but differences in SHBG and OR’s for PCOS remained similar, although confidence intervals were wider (Supplementary Table 1, see section on supplementary materials given at the end of this article). In addition, we excluded those subjects who had given birth within 6 months before participating in this study (two subjects born VMPT, three born LPT and six controls). The results remained similar (Supplementary Table 2).

## Discussion

We found that women born VMPT had higher serum total testosterone, while women born VMPT and LPT had lower SHBG levels and higher FAI. The differences were larger between the VMPT group and the controls than between the LPT group and the controls, which are consistent with a dose-response relationship between gestational age at birth and hormonal levels. In addition, women born LPT had an elevated risk of having PCOS according to clinical and biochemical signs when adjusted for parental and perinatal factors and/or current characteristics.

In a previous study, women born with VLBW had similar levels of reproductive hormone levels compared with their controls at 26–28 years of age (14). However, the number of subjects was small (24 VLBW women, 25 controls). Also elevated adrenal androgen levels have been reported in women born before 32 GW particularly in those born SGA, but testosterone levels were not reported (15). Women born SGA (but not preterm) had a higher prevalence of hyperandrogenism and/or PCOS (11, 12). In the present study, the overall prevalence of PCOS according to the clinical and biochemical signs was 8.8% (5.4% of women in the early preterm group, 12.6% in the late preterm group, and 7.6% in the control group); this is in line with a prevalence of a 6–18% of PCOS as reported in other populations (6, 7, 8). Our results are also in line with the data of the NFBC1966 study in the same area, in which isolated oligoamenorrhea at age 31 years was reported by 11.2%, isolated hirsutism by 10.9%, and both symptoms by 4.2% of the women who returned the questionnaire (26, 27).

As expected, the use of hormonal contraception was associated with a less hyperandrogenic hormonal profile (lower total testosterone levels and FAI and higher SHBG levels) in the linear regression models. However, differences in the use of hormonal contraception between groups were small and not statistically significant, and adjusting for this parameter did not alter the results. We also performed a sensitivity analysis excluding women who used hormonal contraception, in which differences in testosterone concentration and FAI attenuated. However, as 40% of women used hormonal contraception, the power of this analysis was limited and the results should be treated with caution.

### Mechanisms

We have previously shown in this cohort that young adults born VMPT and LPT had higher rates of obesity and higher waist circumferences and body fat percentages than their peers born at term, although this was not statistically significant in this sample (5). Adjustment for body fat percentage attenuated our main findings, and a larger body fat percentage among women born preterm could be among the underlying mechanisms.

Young adults born preterm have lower insulin sensitivity than their peers born at term (1, 3, 4). In the whole population of the ESTER Preterm Birth Study (including men), especially LPT birth was associated with decreased insulin sensitivity (5); this was mainly explained by increased body fat (5). Similarly, in the present study including only women, those who were born LPT were more hyperinsulinemic (higher mean fasting and 2-h insulin levels in the oral glucose tolerance test) and insulin resistant (higher HOMA-IR) than women born at term. Women with PCOS exhibit peripheral insulin resistance (28) and compensatory hyperinsulinemia, both of which are central pathogenetic features of the syndrome (29). Insulin resistance might contribute to hyperandrogenism by several mechanisms: insulin acts synergistically with luteinizing hormone to improve androgen production, and high levels of insulin reduce circulating SHBG levels, thereby increasing the bioavailability of testosterone. In contrast, increasing evidence suggests that an excess of androgen might also contribute to insulin resistance by favoring a predominantly abdominal distribution of body fat and visceral adipose tissue dysfunction (30). Furthermore, abdominal adiposity and adipose tissue dysfunction may induce insulin resistance, and compensatory hyperinsulinemia favors further androgen excess, closing a vicious circle that contributes to the development of the metabolic and cardiovascular risks typically observed in women with PCOS. In line with this hypothesis, in the present study, LPT women exhibited significantly higher waist circumferences compared with controls despite having similar BMIs.

In the present study, neither low birth weight s.d. score nor being SGA explained the difference in clinical and/or biochemical signs of PCOS between women born preterm and women born at term. The exposure to maternal GDM in the present sample predicted lower total testosterone and lower SHBG levels, and maternal type 1 or 2 diabetes predicted PCOS according to clinical and biochemical signs in women, but this did not explain the difference between women born preterm and their peers born at term. Lower SHBG levels were also found in a parallel study that focused on maternal GDM as exposure and included predominantly adults born at term (31). That study showed no difference in total testosterone and did not assess clinical signs of PCOS. It is possible that the association between maternal GDM and hyperandogenemia in the present study is specific to our sampling frame focusing on adult outcomes of preterm birth.

The development of the hypothalamus-pituitary-gonadal-axis in preterm infants is not fully understood. Girls born preterm have highly elevated FSH and LH levels and immature ovarian hormone synthesis during early infancy (32) suggesting disturbance of development of HPG-axis. The origin of PCOS is multifactorial, but there is increasing evidence of a strong genetic background (33). It has been suggested that some genetic determinants are associated with preterm birth as well as with cardiovascular diseases: women with a history of preterm delivery have an elevated risk of cardiovascular disease (34). Also women with PCOS are at greater risk for preterm delivery and have more pregnancy disorders such as pregnancy-induced hypertension, pre-eclampsia, and GDM (35). In total, our findings support the theory that early life factors may be associated with the development of PCOS in later life.

### Limitations

The number of participants in the VMPT group resulted in limited power for the analysis of dichotomous variables (PCOS according to clinical and biochemical signs). Therefore, our findings of no difference in the presence of PCOS between the VMPT group and the controls should be interpreted with caution. Other limitations include the definition of PCOS according to clinical and biochemical signs. Hirsutism should ideally be quantified according to the modified Ferriman–Gallwey score as per common guidelines. However, this scoring was not performed during the extensive clinical examination of the cohort, which included a wide range of reproductive, cardiometabolic, mental health and functional outcomes. Hirsutism is subjective and may be easily over-reported. Nevertheless, we have previously shown that self-reported isolated hirsutism does correlate with increased androgen secretion; further, self-reported oligoamenorrhea and hirsutism can identify women with the typical endocrine and metabolic profiles of PCOS (22, 36). We did not perform ultrasonography to assess the presence of polycystic ovaries (PCO). Women with PCO who might be symptom-free would exhibit milder hormonal and metabolic disorders than symptomatic women and they have been reported to show similar metabolic status than control healthy women (37). We would, therefore, expect that the differences between the clinical and biochemical signs of PCOS and control groups would have been similar or even greater if we would have been able to exclude women with PCO only.

### Significance of the findings

The evidence of the association of preterm birth and CVD risk factors in adulthood is strong and consistent. Although metabolic and cardiovascular disturbances (including abdominal adiposity, disorders of glucose regulation, dyslipidemia, metabolic syndrome, hypertension and cerebrovascular disease) are frequent in adolescent and adult women with PCOS (28, 38, 39, 40), it is still uncertain whether PCOS confers a risk of CVD through mechanisms other than overweight, insulin resistance, diabetes, and metabolic syndrome. However, recent studies indicate that impaired glucose regulation, enhanced ovarian androgen secretion, and chronic low-grade inflammation observed in premenopausal women with PCOS persist after menopausal transition, emphasizing lifelong health risks related to this syndrome (41).

## Conclusion

Women born preterm have more clinical and biochemical signs of PCOS, including hyperandrogenemia, than women born at term. This is consistent with the theory that hyperandrogenemia and PCOS are in part programmed by conditions during early life, and it suggests that these conditions are not limited to those associated with intrauterine growth restriction. Together with increased levels of other cardiometabolic risk factors, the findings also argue for an increased risk of cardiometabolic disorders such as type 2 diabetes and coronary artery disease later in life in adults born preterm.

## Supplementary Material

Supplementary Table 1. Mean differences (in percent) for sex hormone binding globulin and testosterone, and odds ratios for self-reported polycystic ovary syndrome according to the clinical and biochemical signs with 95% confidence intervals (95% CI) in women born very or moderately preterm or late preterm compared to controls born full term. User of hormonal contraception excluded.

Supplementary Table 2. Mean differences (in percent) for sex hormone binding globulin and testosterone, and odds ratios for self-reported polycystic ovary syndrome according to the clinical and biochemical signs with 95% confidence intervals (95% CI) in women born very or moderately preterm or late preterm compared to controls born full term. The subjects who had given birth within six months before participating to the study were excluded.

## Declaration of interest

The authors declare that there is no conflict of interest that could be perceived as prejudicing the impartiality of thus study.

## Funding

The ESTER study was supported by grants from the Academy of Finland (SALVE program for 2009–2012 and grants 127437, 129306, 130326, 134791, 263924 and 315690 to E K), the Emil Aaltonen Foundation, the European Commission (Framework 5 award QLG1-CT-2000-001643; to M R J and Horizon 2020 award 733280 RECAP Research for Children and Adults Born Preterm to E K), Norface DIAL 462-16-040 Award PremLife to E K, the Finnish Foundation for Pediatric Research, the Finnish Government Special Subsidiary for Health Sciences (evo), the Finnish Medical Society: Duodecim, the Jalmari and Rauha Ahokas Foundation, the Juho Vainio Foundation, the Novo Nordisk Foundation, the Signe and Ane Gyllenberg Foundation, the Sigrid Jusélius Foundation, the Jenny and Antti Wihuri Foundation, and the Yrjö Jahnsson Foundation. In addition, NFBC 1986 received financial support from the Academy of Finland (grants 175617, 173454, 24300269, and 24300217); EU FP7-ENV-2008-1-226534; USA/NIH/NHLBI 1-R01-HL087679-01; RFP-RM-06-008; NorFA (50847); Thule Institute (50925); Oulu University Hospital, Oulu, Finland (24301140) and the University of Oulu, Finland (24000692, 24500283).
