# Supplementary material for: Clinical and biochemical signs of polycystic ovary syndrome in young women born preterm
Source: Eur J Endocrinol. 2021 Jun 3;185(2):279–88. doi: 10.1530/EJE-20-1462 (PMC8284903; doi:10.1530/EJE-20-1462)
Supplement: Supplementary Table 1. Mean differences (in percent) for sex hormone binding globulin and testosterone, and odds ratios for self-reported polycystic ovary syndrome according to the clinical and biochemical signs with 95% confidence intervals (95% CI) in women born very or moderately preterm or late  [file supplementary_table_1.pdf]

Supplementary Table 1. Mean differences (in percent) for sex hormone binding globulin and testosterone, and odds ratios for self-reported polycystic ovary syndrome according to the clinical and biochemical signs with 95% confidence intervals (95% CI) in women born very or moderately preterm or late preterm compared to controls born full term. User of hormonal contraception excluded.

	Model	VMPT		LPT		Number of cases in the analysis
		Mean difference or OR (95% CI)	P-value	Mean difference or OR (95% CI)	P-value	
Testosterone	1	10.2% (-2.8, 24.9)	0.128	3.3% (-6.9, 14.5)	0.542	217
	2	11.4% (-3.0, 28.0)	0.124	2.0% (-8.7, 13.9)	0.729	202
	3	12.7% (-2.1, 29.6)	0.096	1.6% (-9.0, 13.4)	0.781	195
SHBG	1	-22.5% (-42.1, 4.6)	0.084	-18.6% (-36.0, 3.5)	0.093	217
	2	-24.6% (-45.7, 4.6)	0.090	-20.2% (-38.6, 3.8)	0.092	202
	3	-19.1% (-42.0, 12.9)	0.210	-19.5% (-38.0, 2.5)	0.103	195
FAI	1	42.3% (2.8, 96.8)	0.033	26.9% (-3.0, 65.9)	0.082	217
	2	37.9% (2.2, 114.0)	0.038	27.7% (-5.0, 71.6)	0.104	202
	3	39.3% (-4.6 103.3)	0.019	26.2% (-6.2, 69.7)	0.123	195
PCOS	1	0.67 (0.17, 2.64)	0.570	2.54 (1.01, 6.14)	0.039	231
	2	1.51 (0.33, 7.00)	0.592	3.97 (1.33, 11.85)	0.014	215
	3	1.29 (0.21, 8.02)	0.780	5.33 (1.61, 17.66)	0.006	197

Covariates in linear and logistic regression models:

1) Age and recruitment cohort

2) Variables in Model 1 and parental educational attainment, maternal body mass index, smoking hypertension, pre-eclampsia and gestational diabetes during pregnancy; subject's birth weight standard deviation scores; and parental history of hypertension, diabetes, and myocardial infarction or stroke

3) Variables in Model 2 and body fat percentage, physical activity and smoking

Abbreviations: FAI, free androgen index; LPT, late preterm; OR, odds ratio; PCOS, polycystic ovary syndrome (according to clinical and biochemical signs); SHBG, sex hormone binding globulin; VMPT, very or

moderately preterm.
