# Supplementary material for: Clinical and biochemical signs of polycystic ovary syndrome in young women born preterm
Source: Eur J Endocrinol. 2021 Jun 3;185(2):279–88. doi: 10.1530/EJE-20-1462 (PMC8284903; doi:10.1530/EJE-20-1462)
Supplement: Supplementary Table 2. Mean differences (in percent) for sex hormone binding globulin and testosterone, and odds ratios for self-reported polycystic ovary syndrome according to the clinical and biochemical signs with 95% confidence intervals (95% CI) in women born very or moderately preterm or late  [file supplementary_table_2.pdf]

**Supplementary Table 2.** Mean differences (in percent) for sex hormone binding globulin and testosterone, and odds ratios for self-reported polycystic ovary syndrome according to the clinical and biochemical signs with 95% confidence intervals (95% CI) in women born very or moderately preterm or late preterm compared to controls born full term. The subjects who had given birth within six months before participating to the study were excluded.

| | Model | VMPT | | LPT | | Number of cases in the analysis |
|---|---|---|---|---|---|---|
| | | Mean difference or OR (95% CI) | P-value | Mean difference or OR (95% CI) | P-value | |
| Testosterone | 1 | 13.6%  (2.4, 26.1) | 0.017 | 8.1% (-0.7, 17.7) | 0.073 | 336 |
| | 2 | 18.1% (5.1, 32.8) | 0.005 | 10.4% (0.9, 20.8) | 0.031 | 311 |
| | 3 | 16.2% (3.5, 30.4) | 0.011 | 8.6% (-0.5, 18.6) | 0.064 | 301 |
| SHBG | 1 | -26.6% (-43.1, -5.4) | 0.017 | -20.5% (-35.4, -2.21) | 0.030 | 336 |
| | 2 | -30.7% (-48.1) | 0.013 | -25.4% (-40.2, -6.8) | 0.010 | 311 |
| | 3 | -19.6% (-37.3, 3.1) | 0.086 | -19.5% (-33.3, -2.8) | 0.024 | 301 |
| FAI | 1 | 54.9% (15.3, 107.9) | 0.004 | 36.0% (6.9, 72.9) | 0.012 | 336 |
| | 2 | 70.5% (22.0, 138.2) | 0.002 | 47.9% (14.5, 91.2) | 0.003 | 311 |
| | 3 | 44.4% (8.7, 91.9) | 0.011 | 35.0% (8.8, 67.4) | 0.006 | 301 |
| PCOS | 1 | 1.28 (0.45, 3.61) | 0.646 | 2.84 (1.21, 6.62) | 0.016 | 375 |
| | 2 | 2.54 (0.62, 10.46) | 0.20 | 4.18 (1.55, 11.29) | 0.005 | 328 |
| | 3 | 2.09 (0.43, 10.02) | 0.36 | 5.60 (1.86, 16.91) | 0.002 | 311 |

Covariates in linear and logistic regression models:

1) Age and recruitment cohort

2) Variables in Model 1 and parental educational attainment, maternal body mass index, smoking hypertension, pre-eclampsia and gestational diabetes during pregnancy; subject's birth weight standard deviation scores; and parental history of hypertension, diabetes, and myocardial infarction or stroke

3) Variables in Model 2 and body fat percentage, physical activity, smoking, and hormonal contraception

Abbreviations: FAI, free androgen index; LPT, late preterm; OR, odds ratio; PCOS, polycystic ovary syndrome (according to clinical and biochemical signs); SHBG, sex hormone binding globulin; VMPT, very or moderately preterm.
